# The Use of Porcine Acellular Dermal Matrix in Single-stage, Implant-based Immediate Breast Reconstruction: A 2-center Retrospective Outcome Study

**DOI:** 10.1097/GOX.0000000000001895

**Published:** 2018-08-06

**Authors:** Yew L. Loo, Sajjad Haider

**Affiliations:** From the * Department of Surgery & Interventional Sciences University College London (UCL), London, United Kingdom; † University Hospital of South Manchester (UHSM), Manchester, United Kingdom.

## Abstract

**Background:**

There have been many reported benefits of the usage of acellular dermal matrices (ADMs) in breast reconstruction. ADM reduces the need for rectus abdominis muscle and serratus anterior coverage by providing an inferolateral coverage of the implant/tissue expander. ADM can provide patients with a 1-staged reconstruction. This study was designed to look at the complication rates of 2 major hospitals in the United Kingdom.

**Methods:**

A large, 2-center retrospective cohort review of patients, who underwent implant-based breast reconstruction using Strattice (LifeCell Corp., Branchburg, N.J.) between March 2009 and November 2017, was performed. One-way analysis of variance was done to compare outcome significance between groups. Independent *t* test was performed to compare outcomes of 2 cohorts and regression analysis to include confounding factors using SPSS Statistics for Windows (Version 22.0. Armonk, NY: IBM Corp.).

**Results:**

A total of 450 and 400 breast reconstructions were carried out in University Hospital of South Manchester (UHSM) and Royal Free London Hospital (RFH), respectively. We recorded a total complication of 37.8% in RFH cohort and 28% in UHSM cohort. The seroma rate was 11.25% and 13.33% in the UHSM and RFH cohort, respectively. Other complications reported were major and minor infections, implant loss, skin necrosis, hematomas, and implant exposure. Multiple linear regression analysis reported that confounding factors affected certain outcomes.

**Conclusion:**

Our 2-center complication rates were comparable to other articles. Results were comparable despite being in 2 different breast units in the United Kingdom.

## INTRODUCTION TO STRATTICE

First introduced in 2001, acellular dermal matrix (ADM) has become increasingly popular in primary tissue expander or implant-based breast reconstruction over the past decade.^1–3^ As many as 87% of breast surgeons globally have used ADMs in implant-based breast reconstructions. The use of ADM in immediate breast reconstructions is as high as 56%.^3,4^ Plastic surgeons have begun to use it in secondary and cosmetic breast reconstructions aiming for better cosmetic outcomes and higher patient satisfaction rate.^5–8^

There have been many reported benefits of the usage of ADMs in breast reconstruction. ADM reduces the need for rectus abdominis muscle and serratus anterior coverage by providing an inferolateral coverage of the implant/tissue expander. Risk of implant exposure has also been greatly reduced with more satisfying definition of the inframammary and lateral mammary folds.^9,10^ In addition, the risk of capsular contracture is lower compared with non-ADM cohorts.^10,11^

A variety of studies have been published on human ADM breast reconstruction outcomes and techniques; however, UK legislation prohibits the use of human tissue.^12,13^ Due to this, tissue expander/implant-based breast reconstructions mainly use porcine materials such as Strattice. A study^14^ found no difference in the outcome of human and nonhuman ADMs; however, the cohort was small and follow-up period was limited. We designed this study to formally evaluate the long-term outcome of Strattice use in a large cohort of breast reconstructions in Greater Manchester and Royal Free London Hospital (RFH) to share our experience with the use of Strattice.

## METHODS

A retrospective review was performed of case notes of all patients who underwent implant-based breast reconstruction using Strattice at University Hospital of South Manchester (UHSM) and Greater Manchester and RFH, United Kingdom. All implant-based reconstructions were performed by breast and plastic surgeons at UHSM and RFH. Two breast surgeons and 4 plastic surgeons were involved at UHSM, and 1 breast surgeon and 3 plastic surgeons were involved at RFH. In most cases, mastectomies were performed by breast surgeons and immediate breast reconstruction by a plastic surgeon in a joined case. However, there was an oncoplastic breast surgeon at UHSM who performed both procedures. Patients who underwent immediate implant-based breast reconstruction using Strattice between March 2009 and November 2017 were included in the study.

### Data Extraction

Patients’ data were collected using a standardized study proforma, which included age, comorbidities such as smoking and obesity (body mass index, >30), indication for mastectomy, adjuvant therapies, length of drain in situ, implant size, and presence of complications. Complications included major and minor infections, implant loss, skin necrosis, hematoma, implant exposure, and seromas. Incidences of capsular contracture were not extracted because incidence of capsular contracture was not assessed in this study. Cellulitis was classified under infections. Major infections were defined as infections that resulted in implant loss, whereas minor infections were classified as infections resolved with antibiotics treatment without further interventions. Patients’ smoking status was based on whether they were smoking at the time of follow-up. Indications for mastectomy include prophylactic BRCA (Breast Cancer susceptibility gene) gene carrier, family history, contralateral risk-reducing procedure, oncologic indications, contralateral cosmetic procedure, revision, and delayed breast reconstruction.

Patient selection was nonrandomized and uncontrolled. Patient suitability for single-stage, implant-based breast reconstruction using Strattice was assessed by the operating surgeons and also based on patient’s preference. Exclusion criteria were patients who had 2-staged reconstruction and patients who were younger than 18 years old. Patients were followed up by consultant and registrar grade surgeons in clinic.

### Statistical Analysis

Statistical comparisons of complications in invasive and risk-reducing mastectomy were performed and comparisons between our Strattice data. Statistical comparisons between the 2 cohorts were done using independent *t* test on SPSS. A *P* value of less than 0.05 was considered to be statistically significant. Regression analysis was performed using SPSS to include the effect of all confounding factors on the outcome associated with the use of Strattice.

### Surgical Technique

The surgical technique of immediate breast reconstruction using ADM, Strattice, has been previously described in many articles.^2,7,10,14,15^ All breast cancer patients were discussed in MDT (Multi-disciplinary Team meeting). All patients were given intravenous antibiotics during induction of general anesthesia. Skin incision was usually performed through a nipple removing skin-sparing or inframammary approach. The pectoralis major muscle was then lifted over the underlying chest wall to create a subpectoral pocket for the implant. The inferolateral edge of the pectoralis major muscle was lifted without excessive detachment of the sternal origin of the pectoralis muscle because it may result in medial migration of the implant. The porcine ADM, Strattice, was then soaked in sterile saline for a minimum of 2 washes before using it. The ADM usually measures 8 × 16 cm and shaped before covering the implant at the inferior border of the pectoralis major muscle. Based on the surgeon and patient’s preference, either a round- or teardrop-shaped silicone implant was placed into subpectoral pocket and the inferior border of the pectoralis muscle was sutured to the superior border of the Strattice. The inferior border of the matrix was then sutured at the inframammary and lateral mammary fold using 2.0 polydioxanone (PDS (Ethicon, Somerville, New Jersey)). The incisions were then closed according to the anatomical layers after 2 drains were inserted into the subcutaneous space and deep to the implant in each breast. The Strattice ADM was not palpable in most cases. Drainage was monitored postoperatively. Patients were continued on intravenous antibiotics (Augmentin or Co-amoxiclav) up to 48 hours postoperatively followed by oral antibiotics upon discharge.

## RESULTS

A total of 400 direct-to-implant breast reconstructions were performed on 320 patients at UHSM and 450 breast reconstructions on 350 patients at RFH. Patients between 24 and 75 years old (median, 50 years) who underwent prophylactic, oncologic, revision, and delayed reconstructions were all included in this study. Of 320 UHSM patients, 250 patients (62.5%) were unilateral and 75 (37.5%) were bilateral breast reconstruction. Sixty-three percent were indicated for invasive breast cancer, 22.5% were risk-reducing procedures, 7.5% were revision surgeries, and only 1% were delayed breast reconstructions (Table 1). Hundred patients (31.12%) had adjuvant chemotherapy, 70 patients (21.9%) had neoadjuvant chemotherapy, and a total of 60 patients (18.75%) previously had radiotherapy. Mean follow-up period was 28.9 months (range, 1–60 months). The mean implant volume for single-stage breast reconstructions was 444.5 mL (range, 150–600 mL).

**Table 1.**
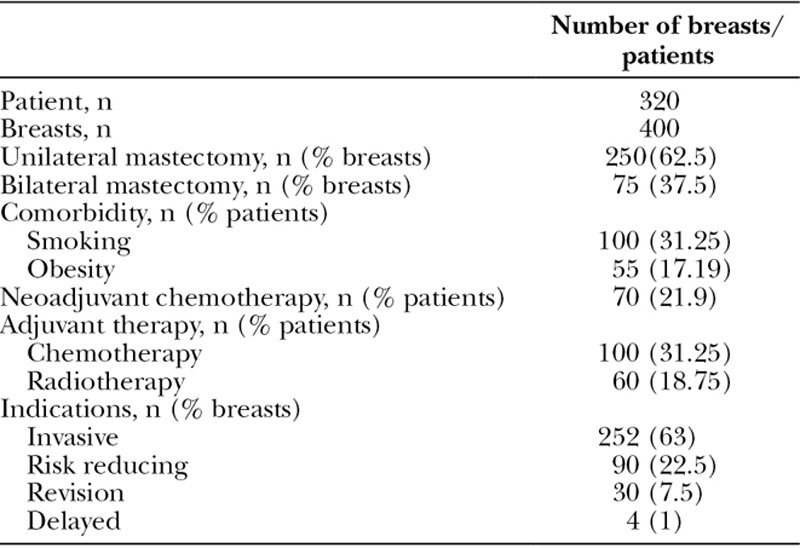
Patient Demographics, Comorbidities, Adjuvant Therapy, and Indication of Procedure for UHSM Cohort

In the Royal Free London (RFH) cohort, 276 patients (61.3%) had unilateral mastectomy and 87 patients (38.7%) had bilateral mastectomy (Table 2). Patients (64.4%) were indicated for invasive procedure, 24.89% were risk reducing, 8.89% were revision surgeries, and 1.78% were delayed breast reconstructions. One hundred fifteen patients (32.9%) had adjuvant chemotherapy, 80 patients (22.9%) had neoadjuvant chemotherapy, and a total of 71 patients (20.3%) previously had radiotherapy. Both cohorts were direct-to-implant breast reconstruction using only Strattice ADM. Mean follow-up period was 32.5 months (range, 1–70 months). The mean implant volume for the breast reconstructions was 434.6 mL (range, 160–700 mL).

**Table 2.**
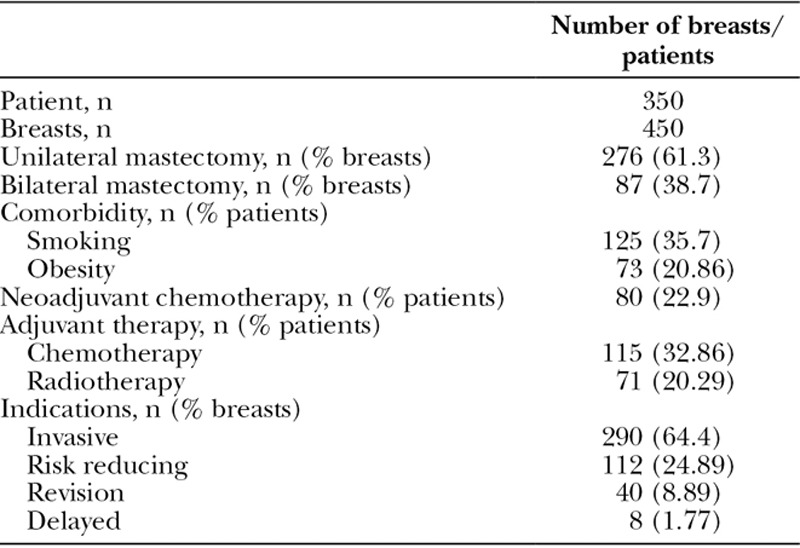
Patient Demographics, Comorbidities, Adjuvant Therapy, and Indication of Procedure for RFH Cohort

The overall complication rate for UHSM cohort was 28% (112 breasts). Forty-five breasts (11.25%) had seroma, 20 breasts (5%) had major infections, 25 breasts (6.25%) had minor infections, 22 breasts (5.5%) had skin necrosis, 15 breasts (15%) had hematomas, 8 breasts (2%) had implant loss, and 5 breasts (1.25%) had implant exposure. The complication rates were further stratified into groups based on indication for surgery. One-way analysis of variance found that there were no significant differences between the outcomes of each group (Table 3).

**Table 3.**
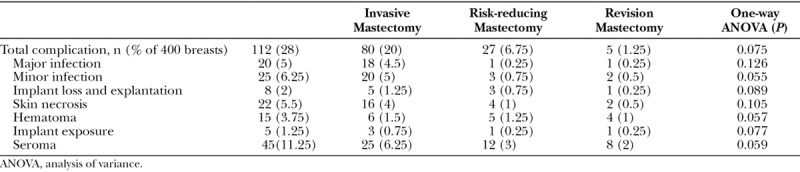
Complications of Strattice-assisted Breast Reconstructions in UHSM Cohort

The overall complication rate for RFH cohort was 37.8% (170 breasts). Sixty breasts (13.33%) had seroma, 30 breasts (6.67%) had minor infections, 24 breasts (5.33%) had major infections, 20 breasts (4.44%) had skin necrosis, 18 breasts (4%) had hematoma, and 8 breasts (1.78%) had implant exposure. One-way analysis of variance did not find any significant difference between outcomes of different surgical indication groups (Table 4).

**Table 4.**
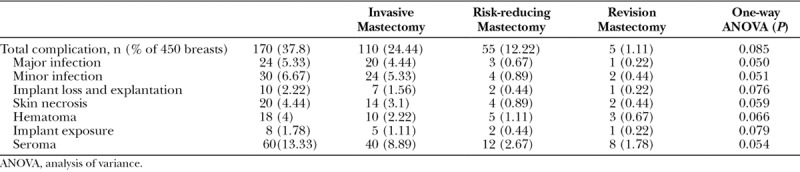
Complications of Strattice-assisted Breast Reconstructions in RFH Cohort P Value <0.05 Indicates Significant Differences

In both centers, all patients had seromas aspirated without any other significant complications. Most of the complications were observed postoperatively and 4 weeks postsurgery at the outpatient clinic. In these patients, there was no evidence of seroma at the time of 6-month follow-up. According to case notes, most patients were discharged at a mean of 3 days (range, 1–14 days). The drains were removed between 6 and 23 days after surgery. In the UHSM cohort, we observed that 15 patients had major or minor infections and 8 patients had skin necrosis during the course of chemotherapy when compared with 25 patients and 14 patients who had major or minor infections and skin necrosis, respectively, in the RFH cohort. With regard to radiotherapy, we found that 5 UHSM patients and 8 RFH patients had skin necrosis after having postoperative radiotherapy. There were no patients lost to follow-up.

Using the independent *t* test, we compared the complications individually between the 2 large cohorts (UHSM versus RFH) and found that there were no statistically significant differences in outcomes between the groups (Table 5). Regression analysis was done as shown in Tables 6 and 7 to include all confounding factors individually and their effects on the outcome of Strattice in both cohorts. In the UHSM cohort, radiotherapy had a significant impact on the rate of minor infections and skin necrosis at *P* = 0.030 and *P* = 0.041, respectively (Table 6). Regression analysis of the RFH cohort revealed statistically significant effects of smoking on seroma rate at *P* = 0.045 and skin necrosis rate at *P* = 0.037. Adjuvant chemotherapy had a significant impact on minor infection rate at *P* = 0.041 and radiotherapy on minor infection and skin necrosis rate at *P* = 0.029 and *P* = 0.032, respectively (Table 7), in RFH cohort.

**Table 5.**
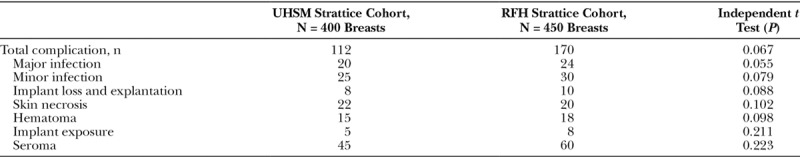
Complications in UHSM Cohort Versus RFH Cohorts P Value <0.05 Indicates Statistical Significance

**Table 6.**
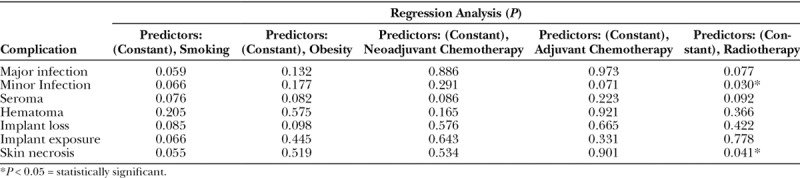
Regression Analysis of Outcome and Confounding Effects on Strattice in UHSM Cohort Using SPSS

**Table 7.**
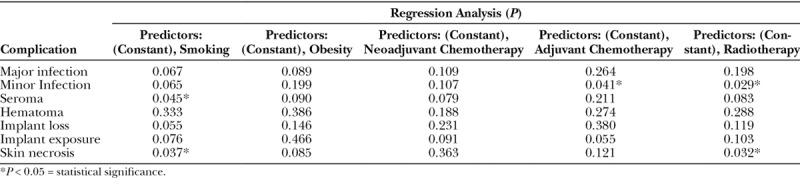
Regression Analysis of Outcome and Confounding Effects on Strattice in RFH Cohort Using SPSS

## DISCUSSION

The use of ADM allows for single-stage breast reconstruction. Although the cost of ADM may be more expensive, fewer clinic visits and revision surgeries are thought to contribute to lesser financial burden.^12^

We observed a total complication rate of 28% and 37.8% with Strattice-assisted breast reconstruction in the UHSM and RFH cohort, respectively. This complication rate was approximately similar compared to a comprehensive literature review done by Krishnan et al^16^ that revealed an average complication rate of 30% in breast reconstruction using ADMs. A review of reports on non-ADM breast reconstruction shows an average complication rate of 34.5%.^16–19^

In a separate report, Castelló et al^19^ reported a high complication rate of 37% in a series of 56 immediate breast reconstructions with anatomic integrated-valve expanders. In contrast to round and Becker expanders that have uniform and softer surface, anatomic expanders have inflation port with a rigid ring on the superoanterior surface, embedded in the body. This rigid ring presses on the mastectomy flap as it inflates. Thus, the higher complication rate may be due to the design of the port.^19^ This can potentially be risky in patients undergoing immediate single-stage reconstruction and in irradiated patients.^20^

According to a pooled analysis of the complications of ADMs in breast reconstructions mainly Alloderm (LifeCell Corp., Branchburg, N.J.),^21^ infection rates were 5.7% and 2% for cellulitis. However, in our study, analysis for infection and cellulitis was performed together. Our major and minor infection rate 5% and 6.25%, respectively, in UHSM cohort and 5.33% and 6.67% in RFH cohort were comparable to the 7.7% infection rate from the meta-analysis in ADM. Another meta-analysis done by Newman et al^22^ reported an infection rate of 5.6% in implant-based breast reconstruction using ADMs. However, they did not clearly define the term ‘‘infection,” that is, whether it included cellulitis or differentiate between infections that were minor (requiring medical management) or major (requiring surgical intervention). A baseline infection rate of 2%–35%^21,23^ in implant-based breast reconstruction without ADM was observed.

Quality of the skin flap intraoperatively was recorded in the operative notes to be in good and acceptable quality having good capillary refill time, color, turgor, and temperature before reconstruction. Yanko et al^20^ reported a skin flap necrosis rate of 18.2% in total of 170 breast reconstruction using anatomic, Becker, and round expanders compared with 4.44% and 5.5% skin flap necrosis rate in UHSM and RFH cohort, respectively. The total complication rate reported was very high at 70.6% (120 breasts). Although their study was based on 3 types of tissue expanders, the number of breasts with complication was significantly higher in the 2-stage tissue expander–based reconstruction. Spear and Majidian^24^ reported a skin flap necrosis rate of 8.0% in their experience with tissue expanders in a series of a 171 breasts reconstruction, whereas Salzberg et al^11^ in his study of 466 breast reconstruction with Alloderm reported a low skin necrosis rate of 1.1% (5 breasts), which is significantly lower when compared with our data.

Our study reported a postoperative hematoma rate of 3.75% and 4% in UHSM and RFH cohort, respectively. A study in 2012 by Salzberg et al^8^ reported a hematoma rate of 1.9% in 105 breast reconstructions with Strattice. A study by Cordeiro and McCarthy^25^ found a hematoma rate of only 0.44% in 2,276 2-stage tissue expander breast reconstructions.

Although our RFH cohort did report significant impact of radiotherapy on incidence of minor infection and skin necrosis, there have not been many articles concluding radiotherapy as a risk of complications such as capsular contracture, infection, and implant failure.^26^ Furthermore, Gunnarsson et al^27^ reported that chemotherapy and radiotherapy did not increase complication rates in Strattice patients, whereas our study showed that adjuvant chemotherapy did increase the risk of minor infection. In our study, most of these patients were found to still be actively smoking at the time of follow-up. Gunnarsson et al^27^ reported smoking as a factor for failure, which is similarly reflected in Table 7. Although we did not assess the incidence of capsular contracture in our study, several studies with a mean follow-up of 6.5–52 months have reported low capsular contracture rate with regard to the use of ADM in immediate breast reconstruction.^28,29,31^

A cost analysis study by Jansen and Macadam^28,29,31^ reported that human ADMs are much more cost-effective when compared with the 2-stage breast reconstruction. The significant cost incurred by the cost of Strattice itself is compensated by the overall reconstruction with fewer revisions and better esthetic outcome.^3^ Because our retrospective cohort study only looked at immediate single-stage breast reconstruction cases, we did not compare the cost-effectiveness with 2-stage expander/implant reconstructions.

One of the main limitations of our study was that there were no records of costs associated with the use of Strattice that were documented at the time of data collection, which made it very difficult for us to perform a cost analysis to further improve our study. Because this was a retrospective cohort study, patient selection was not blinded but selected based on patient’s preference or clinician’s advice, therefore, susceptible to selection bias.

Both of our cohort from Manchester and London reported comparable complication rates when compared with reports on human ADMs and tissue expander–based breast reconstructions. Despite having data collected from 2 different large units in the United Kingdom, our reports showed promising and acceptable results. There is still a need for future studies to compare the cost-effectiveness of single-stage immediate breast reconstruction using Strattice and 2-stage tissue expander–based reconstruction.
